# Comparison of anti-spike IgG, anti-spike IgA levels and neutralizing antibody activity induced by CoronaVac and BNT162b2 vaccines in patients with inflammatory rheumatic diseases receiving immunosuppressive therapy

**DOI:** 10.1186/s41927-023-00342-x

**Published:** 2023-07-19

**Authors:** Fulya Cosan, Ozlem Unay Demirel, Demet Yalcin, Muhammed Mert Sonkaya, Isilsu Ezgi Uluisik, Olida Cecen, Yavuz Furuncuoglu, Deniz Maktav Celikmen, Osman Kara, Erkan Ceylan, Timucin Avsar

**Affiliations:** 1 Faculty of Medicine, Department of Internal Medicine, Division of Rheumatology, Bahcesehir University, Istanbul, Turkey; 2 Faculty of Medicine, Department of Medical Biochemistry, Bahcesehir University, Istanbul, Turkey; 3 Faculty of Medicine, Department of Medical Microbiology, and Infectious Diseases, Istinye University, Istanbul, Turkey; 4 Faculty of Medicine, Department of Internal Medicine, Bahcesehir University, Istanbul, Turkey; 5 Medical Park Goztepe Hospital, Department of Hematology, Bahcesehir University, Istanbul, Turkey; 6 Medical Park Goztepe Hospital, Department of Chest Diseases, Bahcesehir University, Istanbul, Turkey; 7 Faculty of Medicine, Department of Medical Biology, Bahcesehir University, Istanbul, Turkey

**Keywords:** Anti-spike IgG, Anti-spike IgA, BNT162b2, CoronaVac, Immunosuppressive, Neutralizing antibody activity

## Abstract

**Background:**

The importance of COVID-19 vaccination for patients on immunosuppressive (IS) medication has increased due to the high risk of severe disease or mortality. Different vaccines have varying efficacy rates against symptomatic COVID-19, ranging from 46.8% to 95%. The objective of this study was to examine the differences in anti-Spike IgG, anti-Spike IgA, and neutralizing antibody (NAb) activity between the inactive CoronaVac vaccine and the mRNA-based BNT162b2 vaccine in IS patients.

**Method:**

A total of 441 volunteers, including 104 IS patients, 263 healthy controls (HC), who received two doses of CoronaVac or BNT162b2, and 74 unvaccinated patients with a history of SARS-CoV-2 infection, were included in the study. Anti-spike IgG, IgA, and NAb activity were investigated.

**Results:**

Immunogenicity with BNT162b2 was higher than with CoronaVac, but in IS groups, it was lower than HC (CoronaVac-IS: 79.3%, CoronaVac-HC: 96.5%, *p* < 0.001; BNT162b2-IS: 91.3%, BNT162b2-HC: 100%, *p* = 0.005). With CoronaVac, anti-Spike IgG levels were significantly lower than BNT162b2 (CoronaVac-IS: 234.5AU/mL, CoronaVac-HC: 457.85AU/mL; BNT162b2-IS: 5311.2AU/mL, BNT162b2-HC: 8842.8AU/mL). NAb activity in the BNT162b2 group was significantly higher. NAb and anti-Spike IgG levels were found to be correlated. Among the IS group, a significantly lower response to the vaccines was observed when using rituximab. IgA levels were found to be lower with CoronaVac.

**Conclusions:**

Although immunogenicity was lower in IS patients, an acceptable response was obtained with both vaccines, and significantly higher anti-Spike IgG, anti-Spike IgA, and NAb activity levels were obtained with BNT162b2.

**Supplementary Information:**

The online version contains supplementary material available at 10.1186/s41927-023-00342-x.

## Background

The Coronavirus Disease (COVID-19) pandemic has caused high mortality and morbidity worldwide. The control of the pandemic can only be achieved through vaccination. Following the declaration of the COVID-19 pandemic by the WHO in March 2020, vaccine research against COVID-19 was conducted using accelerated procedures under pandemic conditions, and vaccines developed by different research groups received approval [1, 2]. Vaccines created against severe acute respiratory syndrome coronavirus-2 (SARS-CoV-2) infection are basically examined in four groups: DNA or RNA-based vaccines, viral vector vaccines, and inactivated vaccines [3].

Since the vaccines were developed under pandemic conditions with emergency procedures, measuring the effectiveness of the vaccines has become important. Evaluating vaccine effectiveness can involve using symptomatic and/or asymptomatic infection rates as clinical indicators, as well as examining the B and T cell immune responses developed after the vaccine. The fundamental response to the vaccine is immunogenicity, which is defined as the rate of antibody (Ab) formation with the vaccine. Immunogenicity can occur against any protein of the SARS-CoV-2, but the main targets are the spike (receptor binding domain) and nucleocapsid antigens [4, 5]. Various methods are used to evaluate immunogenicity after admisitration of COVID-19 vaccines, and each assay has a different cut-off value for Ab formation. However, there is no clear information about which Ab levels are protective against the disease, and routine evaluation of Ab levels or follow-up after vaccination is not recommended. Most studies on immunogenicity only report on the presence or absence of immunogenicity, and do not provide enough data on how to interpret Ab levels in clinical practice [6–9].

Patients with inflammatory rheumatic diseases (IRD) who use IS drugs are at a higher risk of susceptibility to infection and severe disease compared to the healthy population (HP) [10–12]. Due to the IS treatment, vaccine response as measured by Ab levels against viral particles is expected to be lower in IRD patients than in the healthy controls (HC). Therefore, it is important not only to determine the immunogenicity in the IS group, but also to assess the Ab level and type induced by the vaccine. The effect of different medications on immunogenicity has become an important consideration for choosing treatment strategies, determining the risk, and the need for booster vaccination in these patients.

We aimed to investigate the differences in immunogenicity induced by administration of the inactivated vaccine CoronaVac or the mRNA vaccine BNT162b2 in patients with IRD who use IS drugs, HC, and individuals who have been immunized by infection (INF). Therefore, we evaluated anti-spike IgG, anti-spike IgA, and neutralizing antibody activity (NAb) to determine immunogenicity in the IS, HC, and INF groups.

## Methods

### Study design

In this study, we compared two different vaccine types, CoronaVac and BNT162b2, which have different mechanisms of action and are currently being used in our country. Patients were given the option to choose between the two vaccines for their vaccination, as they were the only available options during the study period.

### Study population

**Fig. 1:**
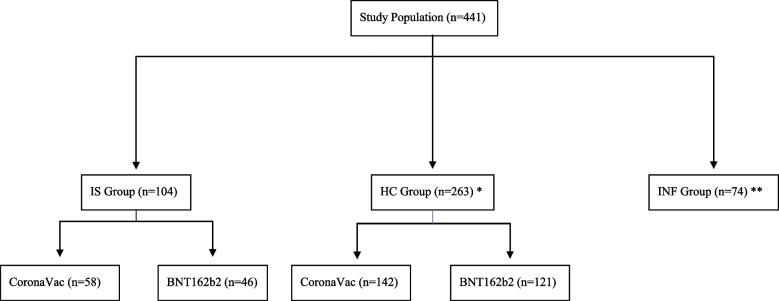
Study Flow chart (IS: Immunosuppressive group, HC: Healthy controls, INF: Infection group) * HC Group: 173 participants included in the study with database screening ** INF Group: 50 participants included in the study with database screening

The study population was divided into three groups consisting of 441 participants aged between 16 and 80 years old: 104 patients on IS medication, 263 HC and 74 individuals with acquired immunity through SARS-CoV-2 infection. A flow chart of the study is illustrated in Fig. 1.

1- IS group: Patients with IRD who have been using steroids or IS drugs with ATC L04 code for at least 1 month and followed up in the rheumatology outpatient clinic are included. Patients receiving hydroxychloroquine (HCQ) monotherapy were not included. However, data on individuals who used HCQ in combination with other drugs were presented in the results section. Patients with IRD who did not use IS drugs were excluded. Two different statistical analyses were performed by including and excluding rituximab using patients, which is a biological agent that directly affects Ab production. The period between the last rituximab administration and inclusion in the study ranged from 6 to 18 months across study participants.

2- HC Group: This group consisted of individuals without known IRD, who did not have a previous SARS-CoV-2 infection or a history of SARS-CoV-2 infection in a first-degree relative, have received two doses of the vaccine, and had Ab tests within two weeks to three months after vaccination.

3- INF Group: Patients who had their first SARS-CoV-2 infection and had Ab tests within two weeks to three months after infection are included.

In IS and HC groups, patients who were vaccinated with two doses and after the second vaccination had their Ab tests within three months were recruited for the study. In the INF group, patients are recruited two weeks after SARS-CoV-2 positivity until three months. People with a history of COVID-19 or febrile infection, known contact with a person with COVID-19, and a history of COVID-19 in their family members were excluded from the study. Detailed inclusion and exclusion criteria are demonstrated in Table 1.

**Table 1:** Inclusion and exclusion criteria

	Definition	Inclusion Criteria	Exclusion Criteria
IS group	Patients taking IS medications who are vaccinated with CoronaVac vaccine or BNT162b2 vaccine	Being between 16 and 80 years old	Taking a break from the IS medication for more than one month
		Taking IS medication due to IRD	Having SARS-CoV-2 infection history
		Not having SARS-CoV-2 infection history and not being contacted with SARS-CoV-2 infected household previously	Being contacted with SARS-CoV-2 infected household previously
		Receiving 2 doses of COVID-19 vaccine and Ab determination in minimum of 2 weeks and maximum of 3 months after the second dose of vaccination	Not receiving the second dose of vaccine
			Ab determination before 2 weeks or after 3 months since the second dose of vaccination
			Having a suspected SARS-CoV-2 infection
			Having a history of both SARS-CoV-2 infection and COVID-19 vaccination
HC group	Those who are vaccinated with CoronaVac vaccine or BNT162b2 vaccine	Being between 16 and 80 years old	Having history of IS disease or IS medication use
		Not having history of IS disease or IS medication use	Having SARS-CoV-2 infection history
		Not having SARS-CoV-2 infection history and not being contacted with SARS-CoV-2 infected household previously	Being contacted with SARS-CoV-2 infected household previously
		Receiving 2 doses of COVID-19 vaccine	Not receiving the second dose of vaccine
		Ab determination in minimum of 2 weeks and maximum of 3 months after the second dose of vaccination	Having a booster dose of the vaccine
			Ab determination before 2 weeks or after 3 months since the second dose of vaccination
			Having a suspected SARS-CoV-2 infection
			Having a history of both SARS-CoV-2 infection and COVID-19 vaccination
INF group	Patients with previous SARS-CoV-2 PCR positivity in three months	Not having history of IS disease or IS medication use	Having history of IS disease or IS medication use
		Not being contacted with SARS-CoV-2 infected household previously	Ab determination before 2 weeks or after 3 months since the PCR positivity
		Ab determination in minimum of 2 weeks and maximum of 3 months after SARS-CoV-2 PCR positivity	Having recurrent SARS-CoV-2 infection

(*IS* Immunosuppressive group, *HC* Healthy controls, *INF* Infection group, *IRD* Inflammatory rheumatic diseases, *Ab* Antibody, *PCR* Polymerase chain reaction)

The IS group is included from the rheumatology outpatient clinic; healthy controls are included from the internal medicine outpatient clinic, and the infection group is included from the Chest Diseases and Internal Medicine clinics.

Since patients who were within the first three months after the second vaccine were included in our study, and lower antibody levels were detected within that vaccine group in individuals whose antibody levels were checked at the third month, the median Ab levels of patients in the first, second, and third months in the study group are presented. Since we do not have measurement data for the same patients’ Ab levels at first, second, and third months, we could not provide data on the rate of Ab level decrease.

The number of patients included in the study has been limited because the enrollment had to be within a certain period (two weeks to three months) after vaccination. To increase the sample size of the control group (HC and INF groups), patients who met the study's inclusion criteria were identified from the hospital's electronic database. The study team collected medical data from individuals who provided consent for research use through the national health data record system (E-nabiz) and patient files. The patients included in the study were required to have anti-Spike IgG testing, which was conducted using the same laboratory and methodology. Additionally, patients in HC and INF groups had no history of chronic inflammatory disease. Information on anti-spike IgG results, COVID-19 vaccination status, SARS-CoV-2 infection history, chronic illnesses, and medication use was collected from identified patients.

### Laboratory methods

The measurement method we used in our study to assess immunity against SARS-CoV-2 is a quantitative IgG assay that targets the spike protein of the virus. We chose to use anti-spike IgG as the primary parameter in our study because previous research has shown that anti-S1 IgG levels are associated with a 72% positive predictive value in high-titer NAbs and a 90.8% negative predictive value in low-titer NAbs [13]. To quantitatively measure IgG Ab levels against the receptor binding domain of the S1 subunit of SARS-CoV-2 in human plasma, we used an automated, two-step chemiluminescent microparticle immunoassay called the SARS-CoV-2 IgG II Quant assay. This assay was conducted using the Abbott Architect ci8200 Autoanalyzer in Abbott Park, Illinois, USA, and the cut-off value was set at 50 AU/mL.

In addition, we investigated NAb activity in 166 patients using the Proteogenix SARS-CoV-2 Surrogate Virus Neutralization Test Kit (sVNT) from Schiltigheim, France.

Mucosal immunity plays a significant role in COVID-19, as the disease can cause infections in both the upper and lower respiratory tracts. Despite this, anti-spike IgA, which is primarily associated with the mucosal immune response, is not commonly measured in clinical practice or research. However, previous research has suggested that IgA is dominant in early neutralization of SARS-CoV-2 [14]. Therefore, we determined the levels of anti-spike IgA Abs in 153 patients using the Mybiosource SARS-CoV-2 Spike S1 IgA ELISA Kit from San Diego, California, USA.

The correlation between IgG, IgA values and Nab activity were evaluated in all groups to assess their potential as predictors and their differences in neutralizing activity at the laboratory level. Studying the correlation between anti-Spike IgG levels and NAb activity can provide important insights into the effectiveness of the immune response in controlling COVID-19. Specifically, it can help determine whether high levels of anti-Spike IgG Abs are predictive of strong neutralizing activity and protection against infection. The correlation between IgG and IgA values was calculated as an additional statistical analysis to compare different Abs based on their source of formation or state of immunosuppression, and to identify any possible relationship.

### Statistical analysis

The SPSS 20 (SPSS Inc., Chicago, IL, USA) program was used for statistical analysis. In the evaluation of study data, descriptive statistical methods (mean, standard deviation, median, frequency, ratio, minimum, maximum) were used in addition to the evaluation of data distribution with Shapiro–Wilk Test. Kruskal–Wallis test was used for comparing three or more groups of quantitative data, and Mann–Whitney U Test was used for comparing two groups. Chi-square analysis was used to determine the relationship between qualitative data. Spearman's correlation analysis was used to determine the relationship between non-normally distributed quantitative data. Multiple linear regression analysis was used to determine the factors affecting the anti-Spike IgG levels and NAb activity. Multiple linear regression analysis performed to determine the effect of administered vaccine types among IS and HC groups. Age and presence of IMID were included in the regression model as potential confounders in terms of NAb activity. The effect has been calculated with a 95% confidence interval in the regression analysis. The significance level was evaluated at *p* < 0.05. Graphics were created using Prism V.8.0 Software (San Diego, California, USA).

### Ethics committee approval

Ethics committee approval was received from Bahcesehir University Ethics Committee (Reference Number: 2021–08/07) and have been performed in accordance with the ethical standards laid down in an appropriate version of the WMA Declaration of Helsinki-Ethical Principles for Medical Research Involving Human Subject. Written informed consent was obtained from all participants.

## Results

The study population comprised of three groups: Patients with immune-mediated inflammatory diseases (IMID) who received either CoronaVac (*n* = 58) or BNT162b2 (*n* = 46) vaccines, a group of HC who received CoronaVac (*n* = 142) or BNT162b2 (*n* = 121) and INF group (*n* = 74). Demographic data for each group is presented in Table 2.

**Table 2:** Characteristics of the patients in IS, HC and INF groups

Groups	Vaccine	N	Sex (Female/Male)	Age (Mean ± SD)	Age Min–Max
IS	CoronaVac	58	43/15	49.5 ± 12.5	(27–86)
	BNT162b2	46	31/15	40.52 ± 11.86	(19–73)
HC	CoronaVac	142	97/45	62.56 ± 16.1	(24–90)
	BNT162b2	121	72/49	38.64 ± 13.63	(18–77)
INF		74	36/38	45.78 ± 11.71	(21–89)

(*IS* Immunosuppressive group, *HC* Healthy controls, *INF* Infection group)

Of 104 IRD patients using IS medications, 61 were diagnosed with rheumatoid arthritis (58.7%), 17 with ankylosing spondylitis (16.3%), 11 with psoriatic arthritis (10.6%) and 14 with other diseases (systemic lupus erythematosus, Behçet's disease, vasculitis etc.) (13.5%). Of these patients, 42 were using methotrexate, 23 were using HCQ, 19 were using leflunomide, 36 were using tumor necrosis factor inhibitors (TNFi), 10 were used rituximab, five were using sulfasalazine, and one was using mycophenolate mofetil.

### Anti-spike IgG: Immunogenicity

**Fig. 2:**
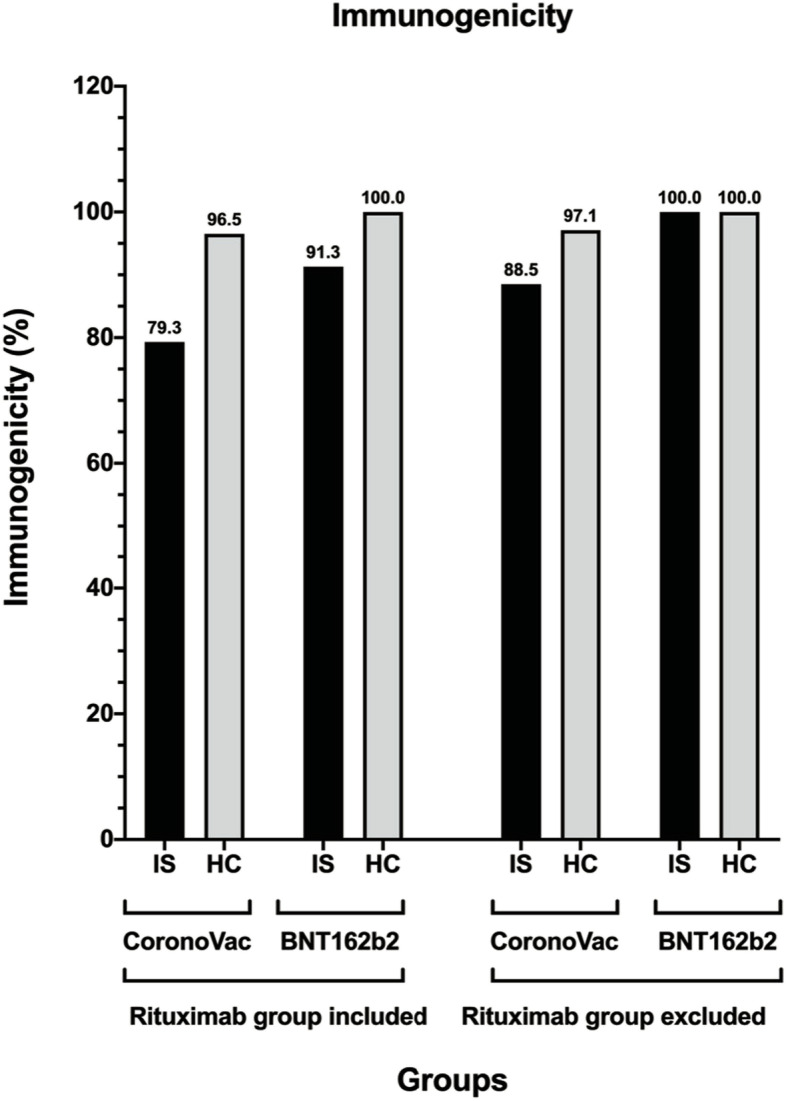
The immunogenicity rates in CoronaVac and in BNT162b2 groups (IS: Immunosuppressive group, HC: Healthy controls)

Immunogenicity was significantly lower in the CoronaVac group among patients with IMID compared to the HC group (79.3% vs 96.5%, *p* < 0.001). Although immunogenicity was higher in the BNT162b2 group, it was still significantly lower in the BNT162b2-IS group than in the BNT162b2-HC group (91.3% vs 100%, *p* = 0.005). When patients treated with rituximab were excluded from the analysis, a significant difference was found between the IS and HC groups in CoronaVac administered patients (88.5% vs 97.1%, *p* = 0.027), while the immunogenicity was 100% in both the IS and HC groups who received the BNT162b2 vaccine (Fig. 2). The data on post-vaccination immunogenicity in patients treated with rituximab is provided in Supplement 1.

### Anti-spike IgG: Ab levels

**Fig. 3:**
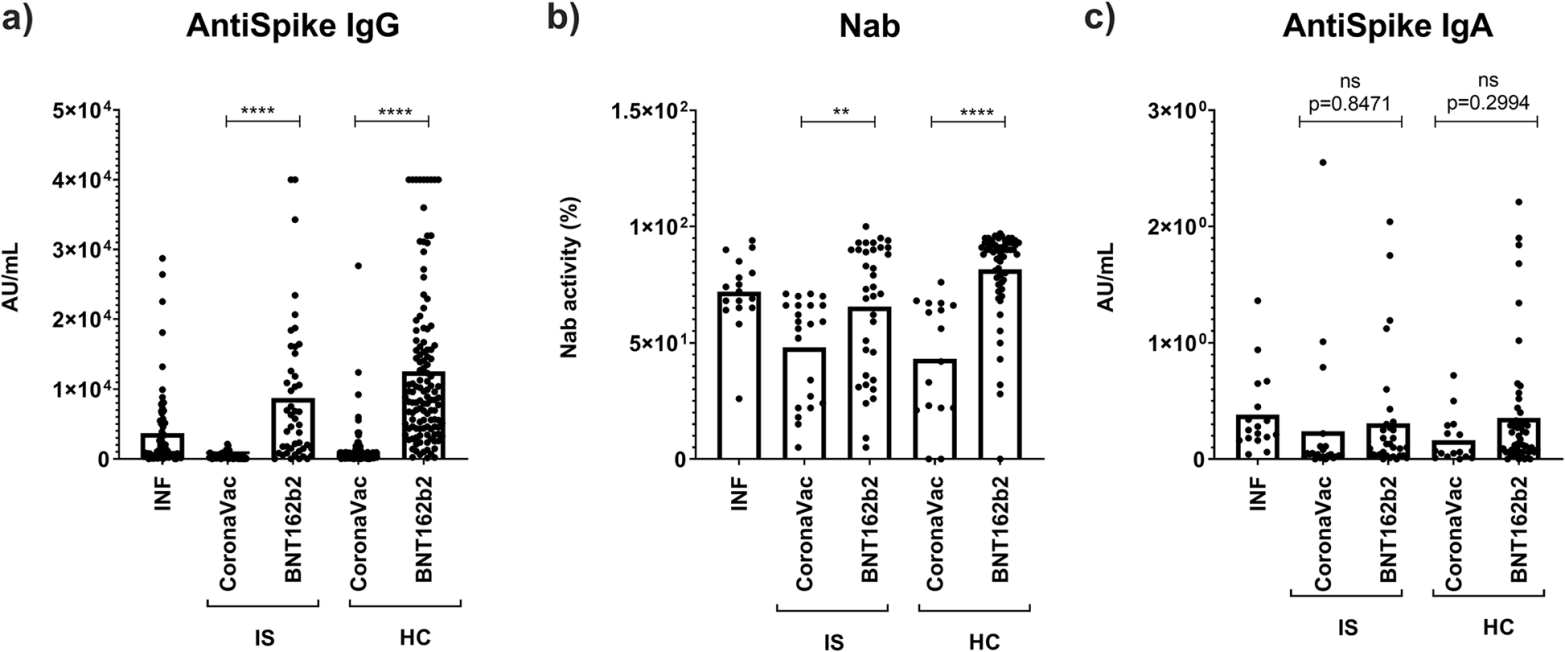
Anti-Spike IgG, anti-Spike IgA and NAb levels by study groups. **a** Anti-Spike IgG levels by study subgroups (median), **b** NAb activity by study subgroups (median), **c** Anti-Spike IgA levels by study subgroups (median) (IS: Immunosuppressive group, HC: Healthy controls, INF: Infection group, NAb: Neutralizing antibody)

The study analyzed the levels of anti-spike IgG Abs, and descriptive data on the Ab measurement results for each study group can be found in Table 3. In the CoronaVac-IS group, the median value of anti-spike IgG was 234.5 AU/mL, while in the CoronaVac-HC group, it was 457.85 AU/mL (*p* = 0.002). Similarly, in the BNT162b2-IS group, the median value of anti-spike IgG was 5311.2 AU/mL, while in the BNT162b2-HC group, it was 8842.8 AU/mL (*p* = 0.007) (Fig. 3). However, when patients using rituximab were excluded, no statistically significant difference was found between the BNT162b2 vaccine administered IS and HC groups (*p* = 0.05) The median anti-spike IgG value in the INF group, as a positive control, was 1283.15 AU/mL. The CoronaVac groups showed lower Ab levels compared to the INF group, while the BNT162b2 groups showed higher Ab levels (Table 4).

**Table 3:** Descriptive data on the Ab measurement results for each study group

		n	Mean ± Sd	Min–Max (Median)
Anti-Spike IgG	IS-CoronaVac	58	401.01 ± 475.49	1.5–2121.5 (234.25)
	IS-BNT162b2	46	8733.18 ± 10,170.45	0.9–40,000 (5311.2)
	HC-CoronaVac	142	1065.54 ± 2722.98	3.6–27,652.4 (457.85)
	HC-BNT162b2	121	12,567.91 ± 11,109.85	126.2–40,000 (8842.8)
	INF	74	3674.51 ± 5722.6	0.5–28,717.9 (1283.15)
Anti-Spike IgA	IS-CoronaVac	35	0.18 ± 0.46	0–2.55 (0.04)
	IS-BNT162b2	32	0.31 ± 0.51	0–2.04 (0.1)
	HC-CoronaVac	16	0.16 ± 0.21	0–0.72 (0.07)
	HC-BNT162b2	53	0.36 ± 0.52	0–2.21 (0.17)
	INF	17	0.38 ± 0.35	0.04–1.36 (0.25)
Neutralizing Ab	IS-CoronaVac	37	45.05 ± 22.03	5–87 (52)
	IS-BNT162b2	35	65.43 ± 28.08	5–100 (73)
	HC-CoronaVac	18	38.33 ± 27.95	0–76 (37.5)
	HC-BNT162b2	59	81.53 ± 18.78	0–97 (90)
	INF	17	71.88 ± 15.74	26–94 (72)

(*IS* Immunosuppressive group, *HC* Healthy controls, *INF* Infection group)

**Table 4:** Comparison of anti-Spike IgG. IgA levels and NAb activity

	Vaccine	Group	Median	P
Anti-Spike IgG level	CoronaVac	IS vs HC (*n* = 58 vs 142)	234.25 AU/mL vs 457.85 AU/mL	0.002^*^
		IS vs INF (*n* = 58 vs *n* = 74)	234.25 AU/mL vs 1285 AU/mL	0.000^*^
		HC vs INF (*n* = 142 vs *n* = 74)	457.85 AU/mL vs 1285 AU/mL	0.000^*^
	BNT162b2	IS vs HC (*n* = 46 vs 121)	5311.20 AU/mL vs 8842.80 AU/mL	0.007^*^
		IS vs INF (*n* = 46 vs 74)	5311.20 AU/mL vs 1285 AU/mL	0.001^*^
		HC vs INF (*n* = 121 vs *n* = 74)	8842.80 AU/mL vs 1285 AU/mL	0.000^*^
Anti-Spike IgA level	CoronaVac	IS vs HC (*n* = 35 vs *n* = 16)	0.04 ng/mL vs 0.065 ng /mL	0.218
		IS vs INF (*n* = 35 vs *n* = 17)	0.04 ng /mL vs 0.25 ng /mL	0.000^*^
		HC vs INF (*n* = 16 vs *n* = 17)	0.065 ng /mL vs 0.25 ng /mL	0.017^*^
	BNT162b2	IS vs HC (*n* = 32 vs *n* = 53)	0.1 ng /mL vs 0.17 ng /mL	0.236
		IS vs INF (*n* = 32 vs *n* = 17)	0.1 ng /mL vs 0.25 ng /mL	0.019^*^
		HC vs INF (*n* = 53 vs *n* = 17)	0.17 ng /mL vs 0.25 ng /mL	0.133
NAb activity	CoronaVac	IS vs HC (*n* = 37 vs *n* = 18)	52% vs 60.5%	0.421
		IS vs INF (*n* = 37 vs *n* = 17)	52% vs 72%	0.000^*^
		HC vs INF (*n* = 18 vs *n* = 17)	60.5% vs 72%	0.001^*^
	BNT162b2	IS vs HC (*n* = 35 vs *n* = 59)	73% vs 90%	0.006^*^
		IS vs INF (*n* = 35 vs *n* = 17)	73% vs 72%	0.938
		HC vs INF (*n* = 59 vs *n* = 17)	90% vs 72%	0.003^*^

(*IS* Immunosuppressive group, *HC* Healthy controls, *INF* Infection group, *NAb* Neutralizing antibody)

(^*^p < 0.05)

### Anti-spike IgA

In the CoronaVac group, the median level of anti-Spike IgA was 0.065 ng/mL in the HC group and 0.04 ng/mL in the IS group, but this difference was not statistically significant (*p* = 0.218) (Table 4). Similarly, in the BNT162b2 group, the median anti-Spike IgA level was 0.17 ng/mL in the HC group and 0.1 ng/mL in the IS group, but this difference was also not statistically significant (*p* = 0.236). In comparison, the median level of anti-Spike IgA in the INF group was 0.25 ng/mL (as a positive control). The levels of anti-Spike IgA were lower in both the IS and HC groups in the CoronaVac group compared to the INF group (Table 3).

### NAb Activity

The median NAb activity was 52% in the CoronaVac-IS group and 60.5% in the CoronaVac-HC group (*p* = 0.421). In the BNT162b2-HC group, the median NAb activity was 90%, while it was 73% in the BNT162b2-IS group (*p* = 0.006) (Fig. 3). The median NAb activity in the INF group was found to be 72% (Table 3).

### Anti-spike IgG, IgA and NAb relationship

**Fig. 4:**
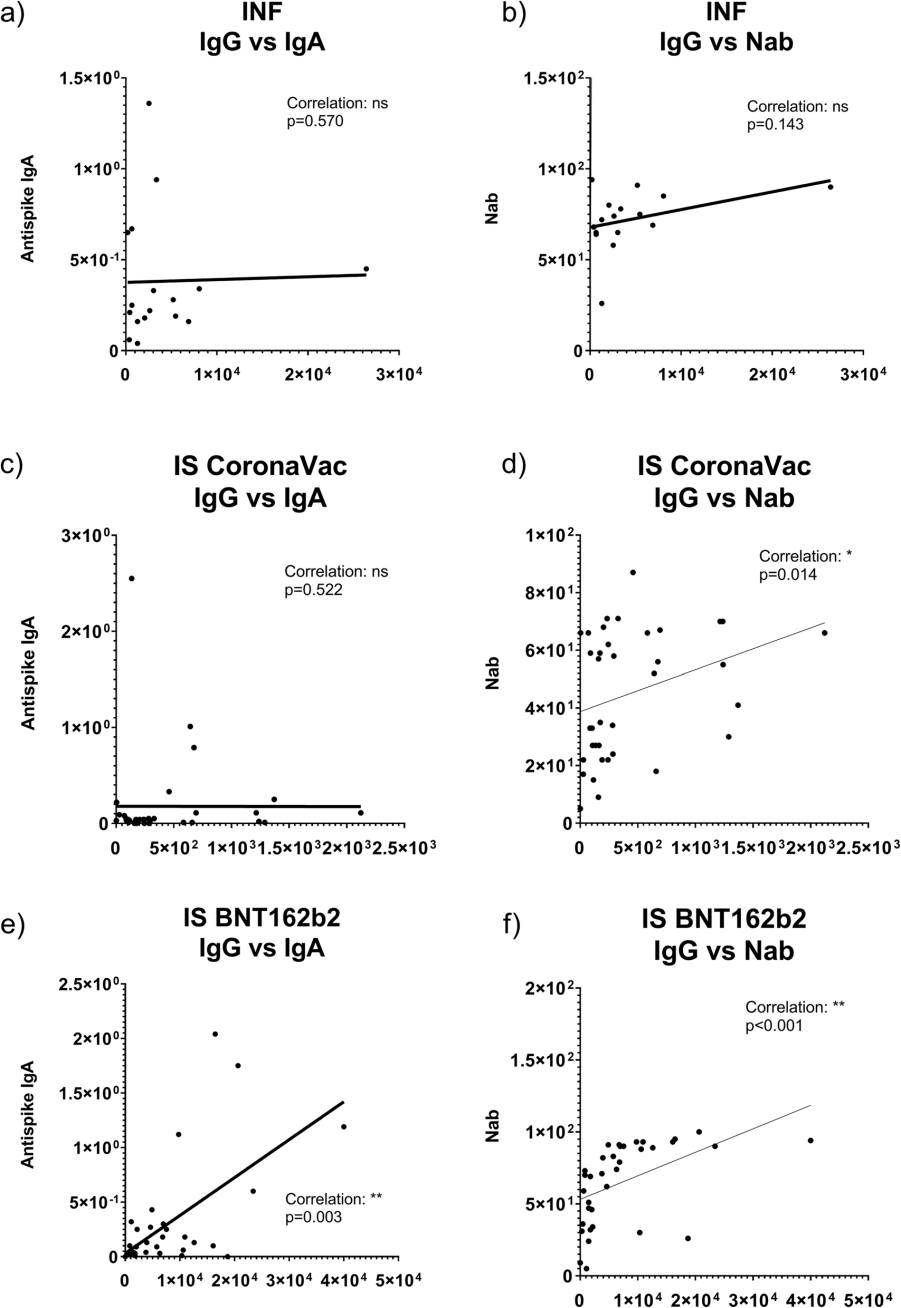
Correlation of anti-Spike IgG, IgA and NAb in IS and INF groups (IS: Immunosuppressive group, INF: Infection group, NAb: Neutralizing antibody)

**Fig. 5:**
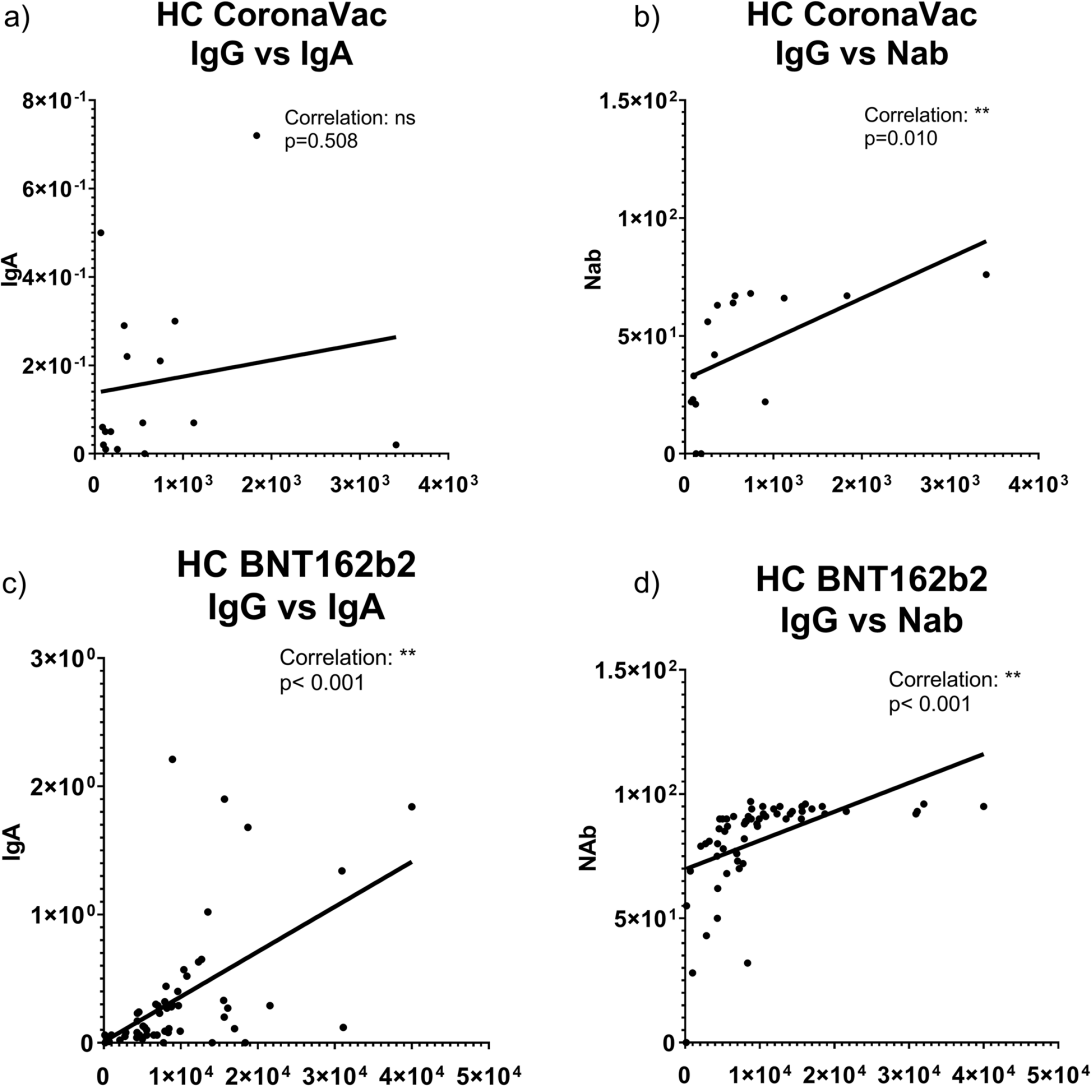
Correlation of anti-Spike IgG, IgA and NAb in HC group (HC: Healthy controls, NAb: Neutralizing antibody)

In the CoronaVac groups, a significant correlation was found between anti-Spike IgG levels and NAb activity, while no correlation was found between anti-Spike IgG and IgA levels, and there was no correlation between anti-Spike IgA levels and NAb activity. In the BNT162b2 group, a significant correlation was found between all three Ab types (IgG, IgA, and NAb) in both the IS (Fig. 4) and HC groups (Fig. 5). The correlation analysis data for all study subgroups are presented in Table 5.

**Table 5:** Correlations between anti-Spike IgG, IgA levels and NAb activity in study groups

	Variable	Anti-Spike IgG level	Anti-Spike IgA level	NAb activity
INF	Anti-Spike IgG level		.570	.143
	Anti-Spike IgA level	.570		.547
	NAb activity	.143	.547	
IS-CoronaVac	Anti-Spike IgG level		.522	.014^*^
	Anti-Spike IgA level	.522		.870
	NAb activity	.014^*^	.070	
HC-CoronaVac	Anti-Spike IgG level		.508	.010^*^
	Anti-Spike IgA level	.508		.841
	NAb activity	.010^*^	.841	
IS-BNT162b2	Anti-Spike IgG level		.003^**^	** < **.001^**^
	Anti-Spike IgA level	.003^**^		** < **.001^**^
	NAb activity	** < **.001^**^	** < **.001^**^	
HC-BNT162b2	Anti-Spike IgG level		** < **.001^**^	** < **.001^**^
	Anti-Spike IgA level	** < **.001^**^		** < **.001^**^
	NAb activity	** < **.001^**^	** < **.001^**^	

(*IS* Immunosuppressive group, *HC* Healthy controls, *INF* Infection group, *NAb* Neutralizing antibody)

^**^Correlation is significant at the 0.01 level (2-tailed)

^*^Correlation is significant at the 0.05 level (2-tailed)

(^***^Spearman correlation test was used for nonparametric variables**)**

### Comparison of CoronaVac and BNT162b2 vaccines

**Fig. 6:**
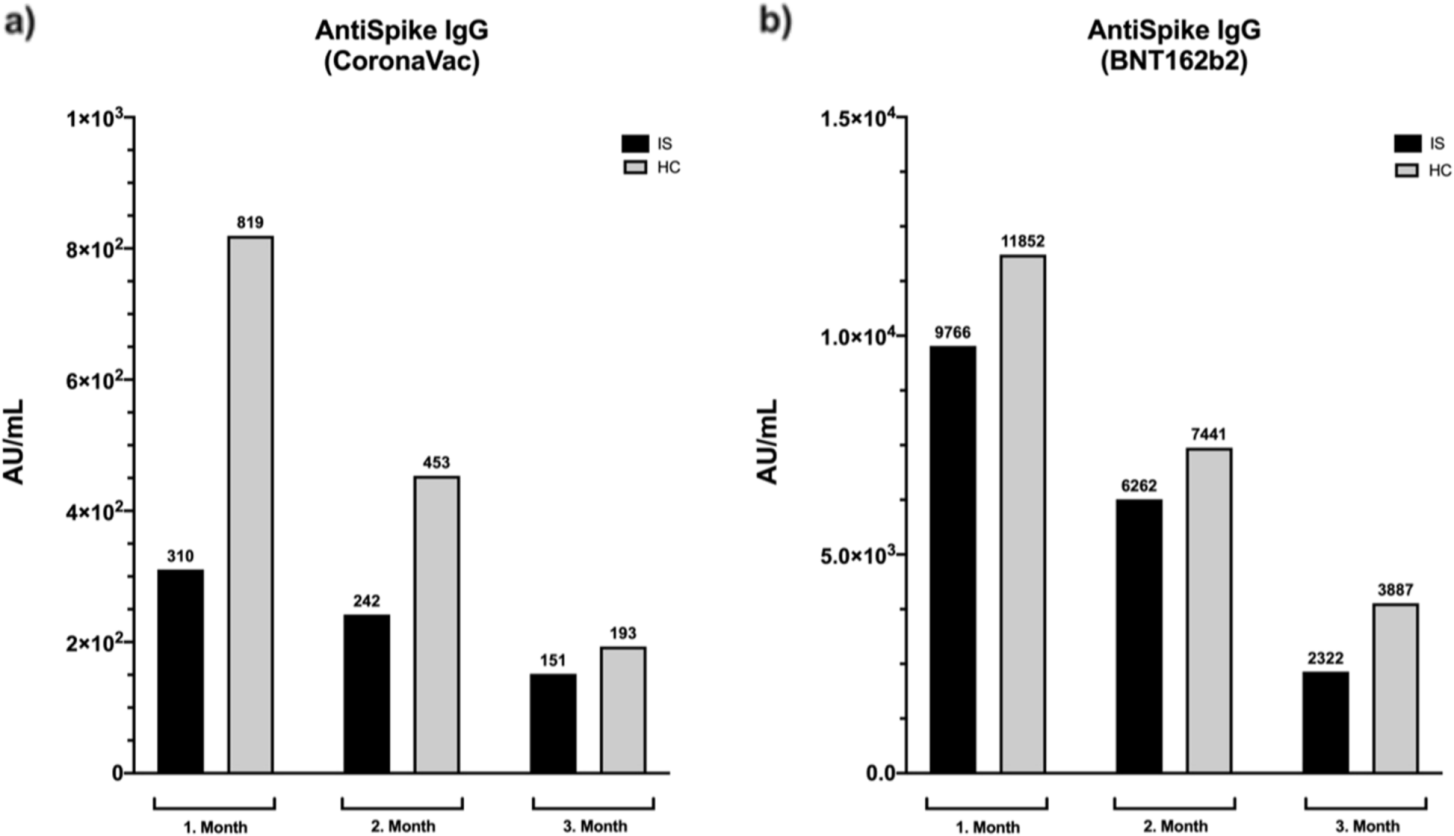
Distribution of anti-Spike IgG medians by months in CoronaVac and BNT162b2 groups (*median anti-Spike IgG levels were shown) (IS: Immunosuppressive group, HC: Healthy controls)

In the IS group, the BNT162b2 vaccine induced higher levels of anti-Spike IgG compared to CoronaVac (median: 5311.2 AU/mL vs 234.25 AU/mL; *p* = 0.000) (Table 4). In the HC group, much higher levels of anti-Spike IgG Ab were observed in the BNT162b2 group compared to the CoronaVac group (median: BNT162b2-HC: 8842.80 AU/mL, CoronaVac-HC: 457.85 AU/mL; *p* = 0.000). When anti-Spike IgG levels were analyzed by months, a gradual decrease in Ab levels over time was observed. The distribution of anti-Spike IgG results by months showed that the Ab levels in the third month in the BNT162b2 group were higher than the CoronaVac group in both the IS and HC groups (third-month median anti-spike IgG: BNT162b2-IS: 2322.4 AU/mL, BNT162b2-HC: 3886.6 AU/mL, CoronaVac-IS: 151.4 AU/mL, CoronaVac-HC: 193.2 AU/mL) (Fig. 6).

CoronaVac and BNT162b2 vaccines were compared in terms of anti-Spike IgA level formation, significantly higher IgA levels were found in the BNT162b2-HC group (median: CoronaVac-IS: 0.04 ng/mL, BNT162b2-IS: 0.1 ng/mL, *p* = 0.029 and CoronaVac-HC: 0.07 ng/mL, BNT162b2-HC: 0.17 ng/mL, *p* = 0.046). In the IS group, anti-Spike IgA levels with the CoronaVac vaccine were found to be lower than the INF group. Statistically significant difference was not found between the INF group and the BNT162b2 vaccinated group in terms of IgA levels (Table 4).

### The effect of independent variables on anti-spike IgG and NAb activity

In terms of anti-Spike IgG levels, results of the multiple linear regression analysis to determine the effect of administred vaccine types among IS or HC groups was found to be statistically significant (F = 52.633, *p* < 0.001) (Table 6). There is a positive and moderately significant relationship between administred vaccine types among IS or HC groups and anti-Spike IgG (R = 0.571, *p* < 0.001). The independent variables included in the model explain 32.6% of the total variance in the effect of anti-Spike IgG (*p* < 0.01).

**Table 6:** Results of multiple regression analysis for predicting anti-Spike IgG with independent variables

**Model**	**Variables**	**Univariable**					**Multivariable**				
		**B**	**Std. Error**	**Standart (B)**	***t***	***p***	**B**	**Std. Error**	**Standart (B)**	***t***	***p***
1	IS-CoronaVac	-5723.43	1209.143	-0.220	-4.733	0.001^**^	-3273.497	1271.378	-0.126	-2.575	0.001^**^
	IS-BNT162b2	3752.937	1358.884	0.131	2.762	0.001^**^	5058.673	1361.174	0.176	3.716	0.001^**^
	HC-CoronaVac	-6351.237	843.852	-0.338	-7.526	0.001^**^	-2608.969	1039.404	-0.139	-2.51	0.001^**^
	HC-BNT162b2	9917.273	810.895	0.504	12.230	0.001^**^	8893.406	1069.859	0.452	8.313	0.001^**^

(*IS* Immunosuppressive group, *HC* Healthy controls)

^**^p < 0.01

^*^p < 0.05

When regression coefficients are examined, it can be seen that the CoronaVac-IS (β = -0.126, *p* < 0.01) and CoronaVac-HC (β = -0.139, *p* < 0.01) variables have a negative effect on the anti-Spike IgG, while BNT162b2-IS (β = 0.176, *p* < 0.01) and BNT162b2-HC (β = 0.452, *p* < 0.01) have a positive and significant effect on it.

Multiple linear regression analysis performed to determine the effect of administred vaccine types among IS or HC groups, age and presence of IMID on NAb activity was found to be statistically significant (F = 28.750, *p* < 0.001) (Table 7). There is a positive and moderately significant relationship between administred vaccine types among IS or HC groups, age, presence of IMID variables and NAb activity (R = 0.589, *p* < 0.001). The administred vaccine types among IS or HC groups, age and presence of IMID variables included in the model explain 34.7% of the total variance in the effect of NAbs (*p* < 0.01)0.2

**Table 7:** Results of multiple regression analysis for predicting neutralizing antibodies with independent variables

**Model**	**Variables**	**Univariable**					**Multivariable**				
		**B**	**Std. Error**	**Standart (B)**	***t***	***p***	**B**	**Std. Error**	**Standart (B)**	***t***	***p***
1	IS-CoronaVac	-24.806	4.800	-0.374	-5.168	0.001^**^	-22.484	4.852	-0.339	-4.634	0.001^**^
	HC-CoronaVac	-29.160	6.543	-0.329	-4.456	0.001^**^	-29.205	6.169	-0.329	-4.734	0.001^**^
	HC-BNT162b2	26.675	3.990	0.463	6.686	0.001^**^	13.987	4.291	0.243	3.26	0.001^**^
	Age	11.349	3.178	0.269	3.571	0.001^**^	3.227	3.335	0.076	0.968	0.335
	Presence of IMID	-16.552	4.150	-0.297	-.3988	0.001^**^	9.643	6.211	0.167	1.553	0.122

(*IS* Immunosuppressive group, *HC* Healthy controls, *IMID* Immune-mediated inflammatory diseases)

^**^p < 0.01

^*^p < 0.05

When regression coefficients are examined, it can be seen that the CoronaVac-IS (β = -0.339, *p* < 0.01) and CoronaVac-HC (β = -0.329, *p* < 0.01) variables have a negative effect on NAbs, while BNT162b2-HC (β = 0.243, *p* < 0.01) has a positive and significant effect on it. While age and presence of IMID found to have significant effect on NAbs in univariable model (β = 0.269, β = -0.297; *p* < 0.01, respectively), no significant results were found in multivariable model (β = 0.076, p = 0.335; β = -0.167; *p* = 0.122; respectively).

### The effect of IS drugs on Ab levels

**Fig. 7:**
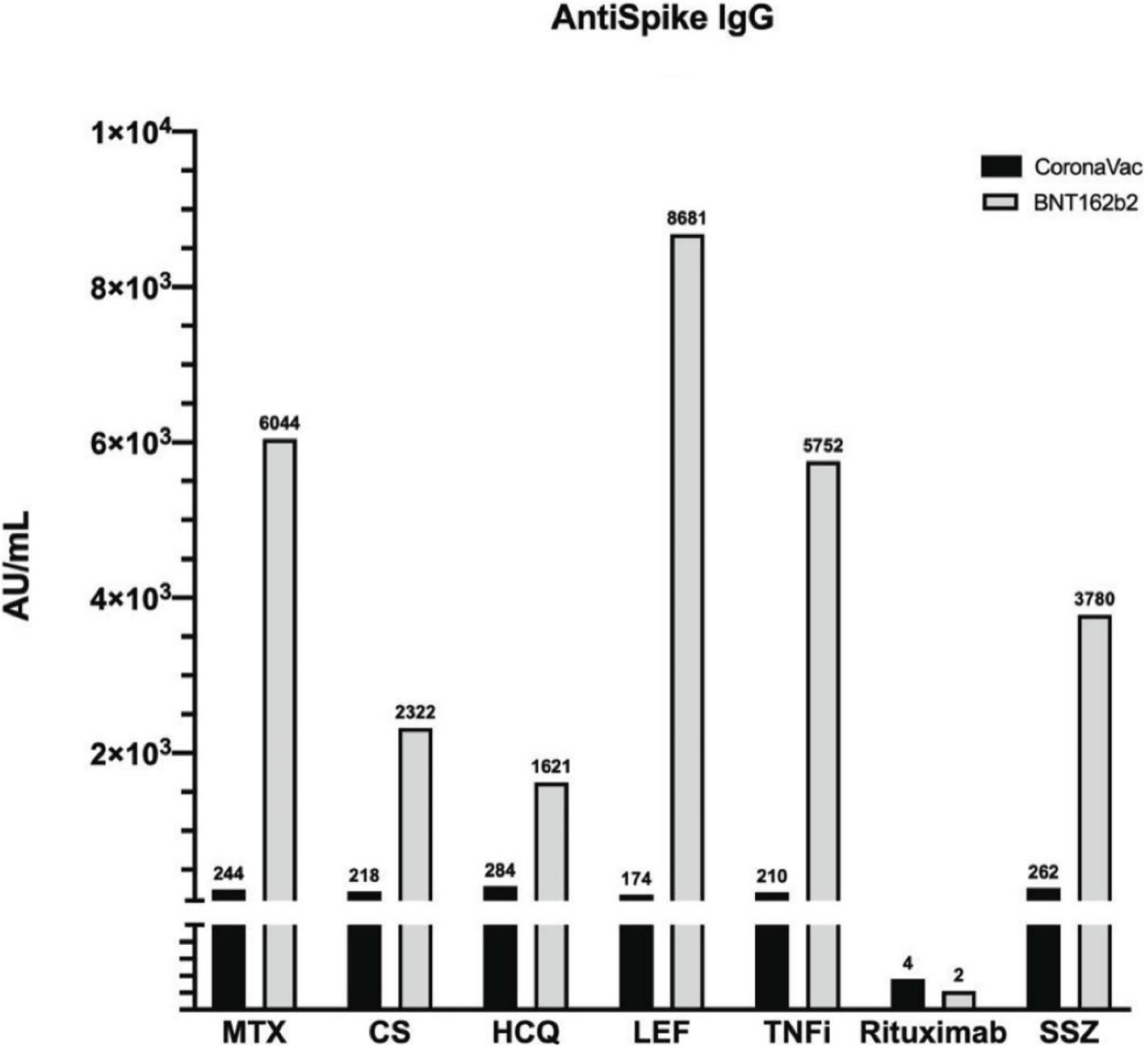
Anti-Spike IgG levels according to different drug use in patients on IS medications by different vaccine groups (* median anti-Spike IgG levels were shown) (MTX: Methotrexate, CS: Corticosteroid, HCQ: Hydroxychloroquine, LEF: Leflunomide, TNFi: Tumor Necrosis Factor inhibitors, SSZ: Sulfasalazine)

When comparing IS IRD patients who received different medications, it was found that those who received corticosteroids, leflunomide, and rituximab had significantly lower anti-Spike IgG Ab responses in the CoronaVac group. Similarly, a significantly lower response was observed in patients using rituximab and HCQ in the BNT162b2 group. Because some of the patients using HCQ were using rituximab concomitantly, it was suspected that the HCQ-related data might be biased. When the analysis was repeated after excluding patients who received rituximab, there were no significant differences in Ab levels in patients treated with HCQ (*p* = 0.425). Figure 7 shows the anti-Spike IgG levels for each drug in both vaccine groups.

**Fig. 8:**
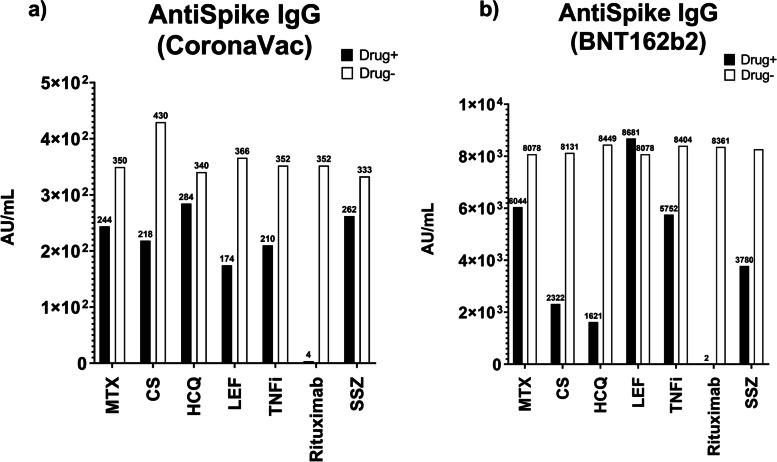
Comparison of anti-Spike IgG levels in CoronaVac and BNT162b2 groups in terms of different drug use (Drug positive or drug negative) (*Study population is evaluated for each drug, according to specific drug use.) (** Median anti-Spike IgG levels were shown.) (MTX: Methotrexate, CS: Corticosteroid, HCQ: Hydroxychloroquine, LEF: Leflunomide, TNFi: Tumor Necrosis Factor inhibitors, SSZ: Sulfasalazine) **a** Comparison in CoronaVac group **b** Comparison in BNT162b2 group

When patients in the IS group were separately evaluated according to their drug use, the comparison data of anti-Spike IgG Ab titers for those using specific drugs and the HC group are presented (Fig. 8) (Supplement 2). However, since patients may be using multiple drugs at the same time, interpreting this data as a direct effect on the Ab levels of the drugs would not be accurate. Nevertheless, the data was presented to demonstrate that the BNT162b2 group achieved high Ab levels in all cases and to show that the use of rituximab resulted in an inadequate Ab response, unlike other IS drug groups. Specifically, among the ten patients who were administered rituximab and had received their last dose at least six months prior, inadequate antibody responses were observed, with levels below 50 AU/ml.

## Discussion

### Immunogenicity of COVID-19 vaccines in HC and IS groups

The immunogenicity after administration of two doses of both BNT162b2 or CoronaVac vaccines in HP was reported to be 100% and 99.5–99.7%, respectively, which is consistent with previous studies [6–8, 15, 16]. In our study, the immunogenicity in the HC group after administration of BNT162b2 or CoronaVac vaccines was found to be 100% and 95.5%, respectively, which is also consistent with the literature.

However, studies investigating patients on IS medications who received the BNT162b2 vaccine have shown lower immunogenicity compared to HP, with rates ranging from 86 to 94% in patients on IS medications and 100% in HC [6–8]. In our study, the immunogenicity of BNT162b2-IS group was found to be 91.3%, and 100% in the HC group (100% vs 100%, when the analysis conducted after excluding rituximab-using patients).

When examining the immunogenicity after the administration of CoronaVac vaccine, a phase 4 trial conducted in patients with IRD found the immunogenicity ratios to be 70.4% and 95.5% in IS and HC groups, respectively [15]. In another study involving patients on IS medications under the age of 60 who received the CoronaVac vaccine, the immunogenicity ratios were found to be 92.7% in the IS group and 99.7% in the HC group [16]. In our study, we observed immunogenicity ratios of 79.3% and 95.5% in the IS and HC groups, respectively.

Previous studies have shown a decline in Ab levels over time [17–19]. In our study, anti-Spike IgG levels were shown on a monthly basis and a significant decrease in Ab levels was found in the third month, particularly in the CoronaVac group compared to the BNT162b2 group. However, since the peak Ab levels in the BNT162b2 group were initially higher, even though they also decreased over time, the Ab levels remained relatively high in the third month.

### Drugs affecting vaccine immunogenicity in IS group

Glucocorticoids, rituximab, abatacept, and mycophenolate mofetil have been reported to negatively affect immunogenicity, with rituximab being the most negatively effective drug according to previous studies [6, 7, 16, 20]. In this study, low levels of Ab levels were found in patients who used rituximab, steroid, and leflunomide in the CoronaVac group, and only rituximab in the BNT162b2 group. There was no statistically significant difference in Ab production between those who used TNFi and those who did not. However, since most of the patients were using combination therapies, it would not be appropriate to attribute low responses to a single drug. Additionally, disease activity status may have been a factor affecting vaccine responses in people using combinations of drugs due to high disease activity. Nevertheless, patients who used rituximab and had passed at least eight months since the last dose did not obtain an adequate Ab response. This finding sets rituximab apart from the other IS agents.

### NAb activity

The primary factors that influence the NAb response against SARS-CoV-2 infection are the duration and severity of the disease. NAb activity levels develop in 40%-70% of patients after infection, and these levels decline within three months [21, 22]. It has also been demonstrated that the induction of NAb activity occurs at varying rates with different vaccines and is closely linked to virus mutations. As a result, assays with different virus targets must be carefully evaluated. [21, 23].

Since the predictive value of NAb activity for immune protection from symptomatic COVID-19 and IgG, IgA values from various disease events were shown, evaluation of Nab activity and antibody titers on correlation with their Nab activity in different groups is worthwhile [24, 25]. A study on the efficacy of the BNT162b2 vaccine in patients on IS medications found that NAb activity levels were correlated with anti-Spike IgG Ab levels [6]. In another study, it was reported that NAb activity levels were 99.5% in controls and 90.5% in people with IMID who received mRNA vaccine. Although a lower Ab response was observed in the IMID group, a high ratio of NAb response was obtained [8]. In a study with patients who received the CoronaVac vaccine, NAb values were 56.3% and 79.3% in the IS and HC groups, respectively [15]. In our study, it was observed that anti-Spike IgG and NAb activity were 90% correlated in the group who received the BNT162b2 vaccine. No correlation was found between anti-Spike IgG levels and NAb activity in the INF group, as it may depend on the severity and duration of the infection. This finding emphasizes the importance of vaccination in individuals who were previously infected, as Abs induced by a mild infection may not have neutralizing activity.

### Anti-spike IgA

Studies investigating anti-Spike IgA in patients who had COVID-19 have shown that IgA response occurs early in the disease, peaks at three weeks, and is stronger and more permanent than the IgM response [26]. Secretory IgA is a potent protector, especially in the mucosal immune response. For this reason, although many vaccine studies target mucosal immunity, it is not yet known to what extent they will be effective. In our study, we observed that IgA levels were lower in the vaccinated group than in individuals who were infected with the virus. The fact that anti-Spike IgA levels acquired by the vaccine are lower than those acquired by infection may explain the occurrence of a mild infection in the upper respiratory tract among vaccinated individuals.

### Inactivated virus vaccine or mRNA vaccine?

In this study, we conducted a real-life comparison of the CoronaVac and BNT162b2 vaccines in IS patients. Although mRNA vaccines have been reported to have very high efficacy, some people preferred the inactivated virus vaccine because they believed it had fewer side effects. However, our findings demonstrate that the immunogenicity after administration of the CoronaVac vaccine is much lower than that of after the BNT162b2 vaccine, and median anti-Spike IgG levels decreases to very low values in the third month after the administration of the second dose. Effective vaccination is especially important in the IS group due to the risks of severe disease, increased hospitalization, and mortality rates. Therefore, we would like to bring to the attention of clinicians that mRNA vaccines yield much higher immunogenicity ratios.

### Limitations

Although we aimed to have a larger study population, we were unable to reach our target due to several reasons. Some patients had already passed the three-month period after vaccination, some had a history of COVID-19, and others had received booster vaccination. As a result, we could only recruit participants who had received two doses of vaccine, did not have a history of infection, and were within three months of their last vaccination within a limited time frame.

After the limited time period, the availability of booster vaccinations, the resurgence of the pandemic, the emergence of new SARS-CoV-2 variants, and other confounding factors made it difficult to recruit patients with the same characteristics, and therefore patient recruitment for the study had to be stopped.

Our study was designed to only include participants who had received two vaccine doses. We excluded individuals who had received booster doses, as the comparison of our defined patient group after booster doses would involve numerous confounding factors. After booster doses were administered, many different groups emerged (3 doses of CoronaVac, 3 doses of BNT162b2, CoronaVac-CoronaVac-BNT162b2, BNT162b2-BNT162b2-CoronaVac), and the time elapsed between the second vaccine dose and booster dose varied among individuals. Additionally, many patients had COVID-19 or had been in contact with an infected individual during the vaccination period or between the two vaccine doses. Most of the patients included in our study declined to receive a booster vaccine dose. Our main goal was to compare the biological effects of the two vaccines with each other. We believe that investigating immune responses with booster doses should be approached as a separate study topic, and a separate study design with a larger patient population under conditions where all confounding factors are nearly equal would be necessary.

One limitation of our study was that we did not have data on pre-vaccination anti-Spike IgG levels. We only excluded patients with a history of COVID-19 or other febrile infections, known COVID-19 contact, or a history of COVID-19 in their family members. This information was verified using the national electronic recording system.

Our original plan was to conduct all three Ab tests for the entire study population. However, due to limited research funding and time constraints, we had to rely on data from the electronic recording system for our control groups (HC and INF groups). Only anti-Spike IgG data was available for this group from the database, whereas all three tests (anti-Spike IgG, IgA, and NAb activity) were performed for the prospective study group.

## Conclusions

There are currently no clinical recommendations or guidelines for using Ab levels to evaluate vaccine response or to make decisions about administering booster doses. In this study, we found that the BNT162b2 vaccine induced higher levels of immunogenicity, anti-Spike IgG, IgA, and NAb activity than the CoronaVac vaccine. Although the immunogenicity ratios in the IS group were lower than in the HC group, they were still at acceptable levels. However, caution should be exercised when using rituximab, as Ab levels were found to be very low in patients using this drug. Additionally, we found a correlation between anti-Spike IgG levels and NAb activity, and observed that vaccine-induced anti-Spike IgA levels were lower than those induced by natural infection. Key points and the study summary for patients are presented in Supplement 3.

## Supplementary Information

**Additional file 1.**

**Additional file 2.**

**Additional file 3.**

## Data Availability

The datasets used and/or analysed during the current study are available from the corresponding author on reasonable request.

## Abbreviations

IS: Immunosuppressive
NAb: Neutralizing antibody
HC: Healthy controls
COVID-19: Coronavirus Disease
SARS-CoV-2: Severe acute respiratory syndrome coronavirus-2
Ab: Antibody
IRD: Inflammatory rheumatic diseases
HP: Healthy population
TNFi: Tumor necrosis factor inhibitors
IMID: Immune-mediated inflammatory diseases
HCQ: Hydroxychloroquine
