# Supplementary figures and images for: Comparison of anti-spike IgG, anti-spike IgA levels and neutralizing antibody activity induced by CoronaVac and BNT162b2 vaccines in patients with inflammatory rheumatic diseases receiving immunosuppressive therapy
Source: BMC Rheumatol. 2023 Jul 19;7:20. doi: 10.1186/s41927-023-00342-x (PMC10355083; doi:10.1186/s41927-023-00342-x)

# Immunogenicity

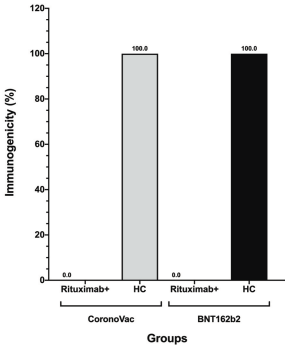

Supplement: Supplementary file 1 — Additional file 1. [file 41927_2023_342_MOESM1_ESM.pdf]
