# Supplementary material for: Comparison of anti-spike IgG, anti-spike IgA levels and neutralizing antibody activity induced by CoronaVac and BNT162b2 vaccines in patients with inflammatory rheumatic diseases receiving immunosuppressive therapy
Source: BMC Rheumatol. 2023 Jul 19;7:20. doi: 10.1186/s41927-023-00342-x (PMC10355083; doi:10.1186/s41927-023-00342-x)
Supplement: Supplementary file 2 — Additional file 2. [file 41927_2023_342_MOESM2_ESM.docx]

Supplement 2: Comparison of anti-Spike IgG levels in individuals with different immunosuppressive drug use and healthy controls

(Mann Whitney U*p<0.05)

|  |  | Anti-Spike IgG |
| --- | --- | --- |
| CoronaVac | MTX | 0.089 |
|  | CS | 0.002* |
|  | HCQ | 0.291 |
|  | LEF | 0.026* |
|  | TNFi | 0.195 |
|  | Rituximab | 0.000* |
|  | SSZ | 0.498 |
| BNT162b2 | MTX | 0.220 |
|  | CS | 0.072 |
|  | HCQ | **0.004*** |
|  | LEF | 0.594 |
|  | TNFi | 0.265 |
|  | Rituximab | 0.001* |
|  | SSZ | 0.148 |
