# Supplementary material for: Comparison of anti-spike IgG, anti-spike IgA levels and neutralizing antibody activity induced by CoronaVac and BNT162b2 vaccines in patients with inflammatory rheumatic diseases receiving immunosuppressive therapy
Source: BMC Rheumatol. 2023 Jul 19;7:20. doi: 10.1186/s41927-023-00342-x (PMC10355083; doi:10.1186/s41927-023-00342-x)
Supplement: Supplementary file 3 — Additional file 3. [file 41927_2023_342_MOESM3_ESM.docx]

**KEY POINTS**

**Evidence before this study**

For patients with inflammatory rheumatological diseases (IRD) who are receiving immunosuppressive (IS) therapy, the humoral immune response to vaccines may be reduced due to the effects of IS therapy. Several studies examining the immunogenicity of COVID-19 vaccines in IRD patients have found that the immunogenicity rates are lower than those in healthy controls (HC), but still within acceptable levels.

However, it is not possible to compare the immunogenicity data of two different vaccines head-to-head since these studies were typically conducted with a single vaccine and in different patient populations at different times using varying methods. Comparing antibody levels between different vaccines is not possible due to the differences in methodology. Previous studies have reported that steroids, mycophenolate mofetil, and rituximab have negative effects on immunogenicity, and vaccine immunogenicity has been shown to decline with age and time.

**Added value of this study**

In this study, we conducted a comparison of the BNT162b2 and CoronaVac vaccines in patients with IRD and HC in a real-life setting. Previous studies only presented data on the presence or absence of immunogenicity, but we further demonstrated and emphasized the differences in antibody levels between the two vaccines. Additionally, we examined the effect of both vaccines on anti-Spike IgA antibody levels and neutralizing antibody activity. To provide a comparison with individuals in the same population who were infected with the same variant of the virus during the same period, we included a non-vaccinated group that had only been immunized by infection as a positive control.

**Implications of all the available evidence and study summary for patients:**

When comparing the immunogenicity, anti-spike IgG, IgA levels, and neutralizing antibody activity rates of the CoronaVac and BNT162b2 vaccines, a much higher level of antibody formation is observed with BNT162b2. It is crucial to have high initial peak levels since antibody levels tend to decline over time. Therefore, vaccines with high efficacy should be preferred in IRD patients due to the increased frequency of the disease and the risk of severe prognosis. Moreover, it is important to consider the very low antibody levels detected with rituximab when choosing this drug for the treatment of IRD. Our study provides insights into the differences between these two vaccines and may help inform vaccine selection for IRD patients.
